# Dietary Supplementation with Ginger (*Zingiber officinale*) Residue from Juice Extraction Improves Juvenile Black Rockfish (*Sebastes schlegelii*) Growth Performance, Antioxidant Enzyme Activity, and Resistance to *Streptococcus iniae* Infection

**DOI:** 10.3390/ani12050546

**Published:** 2022-02-22

**Authors:** Hwa Yong Oh, Tae Hoon Lee, Da-Yeon Lee, Chang-Hwan Lee, Min-Soo Joo, Hee Sung Kim, Kyoung-Duck Kim

**Affiliations:** 1 Department of Marine Biology and Aquaculture, Gyeongsang National University, Tongyeong 53064, Korea; oho1203@gnu.ac.kr (H.Y.O.); dlxogns1204@naver.com (T.H.L.); 2021210237@gnu.ac.kr (D.-Y.L.); dlckdghks25@gnu.ac.kr (C.-H.L.); fkffkxodn@hanmail.net (M.-S.J.); 2 Southeast Sea Fisheries Research Institute, National Institute of Fisheries Science, Tongyeong 53017, Korea; kimkd92@korea.kr

**Keywords:** ginger residue from juice extraction (GRJE), growth performance, antioxidant enzyme activities, resistance against *S. iniae*, black rockfish (*Sebastes schlegelii*)

## Abstract

**Simple Summary:**

Plant-derived feed additives are gaining interest as environmentally friendly and practical alternatives to antibiotics in fish aquaculture. In this study, we evaluated the efficacy of a ginger by-product generated from juice extraction as a feed additive for fish. We compared the effects of varying dietary levels of ginger residue from juice extraction (GRJE) on the growth performance and health status of black rockfish. GRJE diet supplementation had a positive influence on growth, feed utilization, non-specific immunity, and disease resistance and produced no adverse effects. Dietary supplementation of 0.75% GRJE is recommended for improving juvenile black rockfish performance.

**Abstract:**

Plant-derived feed additives provide cost effective and environmentally friendly alternatives to antibiotics for improving fish performance in aquaculture. An 8-week feeding trial was conducted to evaluate the effects of dietary ginger residue from juice extraction (GRJE) on juvenile black rockfish (*Sebastes schlegelii*) growth performance, antioxidant enzyme activities, and resistance to Streptococcus iniae infection. Juvenile rockfish (*n* = 450; initial weight = 2.2 ± 0.01 g) were randomly distributed into 30 L rectangular tanks (30 fish per tank). Five experimental diets with GRJE concentrations of 0% (control), 0.25%, 0.5%, 0.75% and 1% were prepared in triplicate. Three groups of fish were randomly assigned to each diet and fed to apparent satiation twice daily. After the feeding trial, fish were challenged with *S. iniae*, and cumulative survival was observed for six days. Growth parameters, feed efficiency, and the protein efficiency ratio showed a quadratic correlation with the GRJE concentration in the fish diet. Proximate composition and plasma chemistry were not significantly affected. Plasma lysozyme, superoxide dismutase, glutathione, and catalase activities linearly increased with increasing GRJE supplementation levels. Moreover, survival in the *S. iniae* challenge test was significantly higher in fish fed diets supplemented with 0.75–1% GRJE. Our findings demonstrated that 0.75% GRJE dietary supplementation enhanced the growth performance, antioxidant activity, and disease resistance of juvenile black rockfish with no adverse effects.

## 1. Introduction

Fish culture is subject to considerable constraints on fish production due to frequent disease occurrence and low survival rates [1]. Fish farmers use synthetic antibiotics or chemotherapy to manage pathogens, reduce stress, improve immunity, and increase fish resistance to disease [2,3]. However, traditional aquaculture management approaches can increase the occurrence of antibiotic-resistant strains, destroy or alter the natural microbial community of the environment, and leave residues in fish products [4,5]. These factors have contributed to an urgent demand for aquaculture production free from antibiotics and chemicals, raising interest in the development of plant-derived feed additives as alternatives to traditional chemotherapy and antibiotics for improving fish growth and immunity [6].

Fish diets can be supplemented with various natural substances containing animal or herbal ingredients, such as levamisole, glucan, vitamin C, and yeast, to strengthen immune function and prevent disease [2]. However, high production costs, unavailability, and short-term effectiveness limit the practical application of these immunostimulants [7,8]. Thus, there is a need to develop plant-derived feed additives that are inexpensive, easy to apply to the feed industry, readily available, and generate stable effects.

In recent decades, many studies have demonstrated the advantages of herbal medicines, including improved sustainability and fewer side effects than other immunostimulants [6]. Among medicinal plants, ginger (*Zingiber officinale* Roscoe) has been widely studied due to its antioxidant [9], antibacterial [10,11,12,13], antiparasitic [14], anti-inflammatory [1], immunostimulatory [15,16,17], and growth-promoting [1,17,18] properties. The benefits of ginger powder, ginger essential oil, and ginger extracts as a diet supplement for fish have been reported [6,19,20,21]. However, the efficacy of using a ginger by-product from juice extraction as a feed supplement for black rockfish (*Sebastes schlegelii*) has not been investigated.

Ginger juice is gaining popularity as a health drink due to its taste and numerous health benefits [22,23]. Ginger residue is generated as a by-product from juice extraction. Ginger residue from juice extraction (GRJE) has been applied as crop fertilizer but is often treated as industrial waste [24]. However, GRJE is also a rich source of potentially valuable bioactive compounds, such as fibers, polyphenols, and flavonoids [23,24]. Furthermore, GRJE was demonstrated to be an effective pathogen inhibitor and potential alternative to antibiotics in pig diets [25,26]. However, GRJE diet supplementation has not yet been indicated as an alternative to antibiotics and/or chemotherapy in aquaculture.

*S. schlegelii* is one of the most important mariculture species in East Asia, including China, Japan, and Korea. Annual rockfish production reached 21,571 metric tons in 2020 in Korea [27]. However, significant mortality rates associated with disease outbreaks caused by three bacteria, *Streptococcus* spp. (Gram-positive), *Photobacterium* spp. (Gram-negative), and *Vibrio* spp. (Gram-negative), have a detrimental effect on black rockfish culture in Korea [28]. The aim of this study was to determine the effects of dietary GRJE supplementation on growth performance, antioxidant capacity, and resistance to *S. iniae* of juvenile rockfish.

## 2. Materials and Methods

### 2.1. Preparation of Ginger Residue from Juice Extraction (GRJE)

GRJE was obtained from Youngjin Healthy Juice Store (Daegu, Korea). Briefly, fresh ginger was subjected to pressurized hot water extraction for 8 h at 80–90 °C. The remaining GRJE was brought to the laboratory and dried for 72 h at 20 °C in a dryer (KED-M07D1, Kiturami Co. Ltd., Seoul, Korea), crushed into a fine powder with a blender, and stored at −20 °C.

### 2.2. Experimental Diet Preparation

Ingredients and nutritional information for the five formulated diets are shown in Table 1. The protein sources in the control diet were 50% pollock meal and 11.5% fermented soybean meal. Fish oil (4.5%) and soybean oil (4.5%) were used as lipid sources, whereas wheat flour (27%) was used as a carbohydrates source. The control (GRJE0) diet did not include GRJE. For the experimental diets, graded levels of GRJE supplementation at 0.25% (GRJE0.25), 0.5% (GRJE0.5), 0.75% (GRJE0.75), and 1% (GRJE1) were included at the expense of an equal amount of wheat flour. The feed ingredients were physically combined with the oils and distilled water in a mixer, and the dough was extruded into 3–5 mm diameter pellets using a laboratory pellet extruder (SL Machinery, Incheon, Korea). The experimental pellets were dried at a temperature of 20 °C for 48 h in a dryer (KED-M07D1, Kiturami Co. Ltd., Seoul, Korea) and then frozen at a temperature of −20 °C until use.

### 2.3. Experimental Fish and Conditions

Juvenile rockfish was obtained from a local fish farm (Namhae-gun, Gyeongsangnam-do, Korea) and transferred to the Marine Bio-Education and Research Center (Tongyeong, Gyeongsangnam-do, Korea) in 5-ton circular tanks equipped with sufficient aeration and seawater. For two weeks, fish were adapted to the experimental settings by being fed a commercial feed (Jeil Feed Co., Gyeongsangnam-do, Korea). Juvenile rockfish (*n* = 450; mean ± SD body weight = 2.2 ± 0.01 g) were randomly distributed into 15 30 L flowthrough tanks (water capacity, 25 L; 30 fish per tank) with seawater flow (1.2 L/min) and aeration. Three tanks were assigned to each experimental group. For 8 weeks, triplicate groups were fed the experimental diets twice daily (09:00 h and 17:00 h) until visible satiation. Feed consumption was recorded daily for each tank. The mean water temperature, salinity, and dissolved oxygen during the experimental period were 21.2 ± 0.22 °C, 30.13 ± 0.12 psu, and 7.0 ± 0.06 mg/L, respectively. The photoperiod followed natural conditions.

### 2.4. Bacterial Challenge Test

After the feeding trial, 10 fish were randomly selected from each tank and redistributed into 15 30 L tanks for the challenge test. The Korean Culture Collection of Aquatic Microorganisms, National Institute of Fisheries Science (Busan, Korea) provided the pathogenic bacteria, *S. iniae*. Then, 0.1 mL of *S. iniae* culture suspension (5.0 × 10^6^ CFU/mL) was intraperitoneally injected into each fish. The water temperature and dissolved oxygen were maintained at 20.5 ± 0.15 °C and 6.8 ± 0.25 mg/L, respectively. Cumulative mortalities were recorded daily for six days.

### 2.5. Sample Collection

After the feeding trial, the surviving fish were counted and starved for 24 h before the total biomass was recorded to calculate growth performance. Ten fish in each tank were randomly selected and anesthetized using 100 ppm tricaine methanesulfonate (MS-222). Using heparinized syringes, blood samples were obtained from the caudal veins of fish. Plasma was collected and kept in a freezer at −80 °C until analysis following centrifugation (7500 rpm for 10 min). After blood sampling, the remaining fish (≥5 fish) in each tank were homogenized and used for whole-body proximate composition analysis.

### 2.6. Analyses

#### 2.6.1. Chemical Analyses

The diets and whole-body of fish were analyzed in accordance with the method of Association of Official Agricultural Chemists methods [29]. The crude protein content was evaluated using the Kjeldahl method with a KD310‐A‐1015 KjelROC Analyzer (OPSIS Liquid LINE, Sweden). The Soxtec extractor (ST 243 Soxtec™; FOSS, Hillerod, Denmark) was used to determine the crude lipid content. The moisture content was assessed by oven drying at 105 °C for 24 h, and the ash content was determined using a muffle furnace at 550 °C for 4 h.

#### 2.6.2. Plasma Biochemical Analysis

The aspartate aminotransferase activity (AST), alanine aminotransferase activity (ALT), total cholesterol (T-CHO), total protein (TP), and glucose (GLU) were all analyzed using an automatic chemistry system (Fuji Dri-Chem NX500i; Fujifilm, Tokyo, Japan).

#### 2.6.3. Lysozyme Activity Analysis

The activity of lysozyme in plasma was measured using a commercial kit (EnzChek™ Lysozyme Assay Kit, E22013, Thermo Fisher Scientific, Waltham, MA, USA). This kit measures lysozyme activity using fluorophore fluorescein incorporated in *Micrococcus lysodeikticus* cell walls, which quenches the fluorescent signal. Plasma was diluted with 25 mL of 1X reaction buffer, which was made up of 0.01 M NaCl and 0.01 mg NaCl. It was then mixed in with 50 mL of fluorescein isothiocynate-labeled *Micrococcus lysodeikticus* (50 mg/mL) in a 96-well plate at 37 °C for 30 min. Fluorescence intensity was measured with a fluorescence reader (1420 Multilabel Counter Victor3, Perkin Elmer, CT, USA) at 485/535 nm. Each value was divided by the amount of fluorescence in the background, which was measured for a no-enzyme control. The lysozyme activity of the samples that were used in the study was determined from a standard curve made with lysozyme from chicken egg whites.

#### 2.6.4. Superoxide Dismutase Activity Analysis

The activity of superoxide dismutase (SOD) was determined according to the manufacturer’s instructions using a Cayman’s Superoxide Dismutase Assay Kit (Cayman Chemical, Ann Arbor, MI, USA). To determine the activity, 10 μL of plasma was introduced to 200 μL of the radical detector, and then they were mixed together. The xanthine oxidase was added to 20 L of the mixture, and it was incubated on a shaker for 20 min to initiate. The spectrophotometer (Thermo Scientific MULTISKAN GO, Vantaa, Finland) was used to measure the absorbance at 440 nm.

#### 2.6.5. Catalase Activity Analysis

The catalase (CAT) activity was analyzed following the manufacturer’s instructions using a Cayman’s Catalase Assay Kit (Cayman Chemical, Ann Arbor, MI, USA). To analze the activity, 20 μL of plasma was mixed with 30 μL of methanol and 100 μL of assay buffer, and then mixed again. The 20 μL of H_2_O_2_ was used to initiate the reaction. The mixture was incubated at room temperature for 20 min. Potassium hydroxide and purpald chromagen were added to the mixture to terminate it, and the mixture was kept at room temperature for 10 min. Finally, 10 μL of potassium periodate was added and shaken for 5 min at room temperature. At 540 nm, the solution’s absorbance was measured using a spectrophotometer (Thermo Scientific MULTISKAN GO, Vantaa, Finland).

#### 2.6.6. Glutathione Activity Analysis

The glutathione (GSH) concentration was determined using a Cayman’s GSH Assay Kit (Cayman Chemical, Ann Arbor, MI, USA). For the analysis, 50 μL of plasma samples in a 96-well plate were mixed with 150 μL of freshly prepared assay cocktail, which contained MES buffer (2-(N-morpholino) ethanesulfonic acid), cofactors, enzymes, water, and DTNB (5,5′-dithio-bis-(2-nitrobenzoic acid)). At 405 nm, the absorbance was measured every 5 min for 30 min using a spectrophotometer (Thermo Scientific MULTISKAN GO, Vantaa, Finland).

### 2.7. Calculations and Statistical Analyses

The following parameters were used to calculate the grow performance of fish:

- Fish at the end of the feeding trial/number of fish at the initial of feeding trial) × 100;
- Weight gain (WG) = (final body weight–initial body weight)/initial body weight;
- Specific growth rate (SGR, %/day) = (ln final weight of fish–ln initial weight of fish)/days of feeding) × 100;
- Feed consumption (g/fish) = total feed intake/number of surviving fish;
- Feed efficiency (FE) = WG/feed consumed;
- Protein efficiency ratio (PER) = WG/protein consumed;
- Protein retention (PR) = Protein gain × 100/protein consumed.

All percentage values were arcsine transformed before statistical analysis. Variable data were checked for normality and homogeneity of variance using the Kolmogorov–Smirnoff and Levene’s tests, respectively [30]. ANOVA was conducted to determine where dietary GRJE concentrations significantly affected the observed response (*p* < 0.05); then, orthogonal polynomial contrasts (linear, quadratic, and cubic) were used to evaluate the response for all dependent variables [31]. The Kaplan–Meier and log-rank and Wilcoxon tests were used to plot the fish survival curves during the *S. iniae* challenge test. SPSS version 25.0 software was used for all statistical analyses (SPSS Inc., Chicago, IL, USA).

## 3. Results

### 3.1. Growth Pergormance

After the 8-week feeding trial, the effects of GRJE supplementation on juvenile rockfish growth performance and feed utilization were determined and are presented in Table 2. Dietary GRJE had no significant effect on SR and FC. However, increasing dietary GRJE levels affected the FBW, WG, SGR, FE, and PER, presenting quadratic trends (*p* < 0.05). The FBW, WG, SGR, FE, and PER were significantly higher in the GRJE0.25, GRJE0.5, GRJE0.75, and GRJE1 treatments than in the GRJE0 treatment (*p* < 0.05).

### 3.2. Whole-Body Chemical Composition

The moisture content of whole-body of fish varied between 72.0% and 72.4%, the crude protein content between 16.6% and 16.9%, the crude lipid content between 5.7% and 5.9%, and the ash content between 4.1% and 4.3%. Graded levels of dietary GRJE exhibited no significant effect on whole-body proximate composition (Table 3).

### 3.3. Plasma Biochemical Parameters

Table 4 shows the effect of GRJE supplementation on juvenile rockfish plasma biochemical parameters. The AST, ALT, T-CHO, TP, and GLU were significantly different among diet groups.

### 3.4. Plasma Lysozyme Activity and Antioxidant Parameters

The effects of GRJE supplementation on plasma lysozyme activity and antioxidant parameters are shown in Table 5. Lysozyme, SOD, and CAT activities and GSH content showed a linearly increasing trend with the increasing dietary GRJE level; lysozyme (*p* < 0.05), SOD, and CAT activities and GSH content were significantly higher in the GRJE0.75 and GRJE1 groups than in the GRJE0, GRJE0.25, and GRJE0.5 groups (*p* < 0.05).

### 3.5. Bacterial Challenge Test

**Figure 1:**
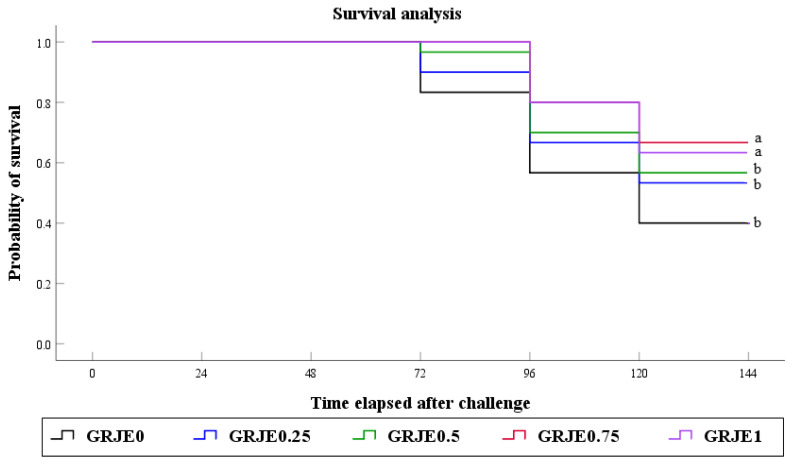
Survival of juvenile black rockfish fed experimental diets with graded levels of GRJE for 8 weeks, then infected by *Streptococcus iniae*. Values are mean ± SE of triplicate groups. Different lowercase letters indicate significant differences (*p <* 0.001; log-rank and Wilcoxon tests). GRJE, ginger residue from juice extraction.

The challenge test with *S. iniae* was conducted after the 8-week feeding trial, and cumulative mortality and survival were recorded over six days. The probability of survival was significantly higher in GRJE0.75 (53.3%) and GRJE1 (46.7%) than GRJE0 (80.0%), GRJE0.25 (66.7%), and GRJE0.5 (66.3%) groups (Figure 1). However, differences in survival among GRJE0, GRJE0.25, and GRJE0.5 groups were not significant.

## 4. Discussion

Supplementing fish diets with various types (powder, extract, oil, by-product, etc.) of ginger (*Zingiber officinale*) has been demonstrated to enhance fish performance [2,16,32,33,34]. In our previous study, we showed that dietary GRJE supplementation significantly improved juvenile black rockfish growth performance, innate immunity, and disease resistance against Gram-negative bacteria *V. harveyi* [34]. However, further investigation was needed to establish the stimulatory dose of GRJE in fish diets. Here, we evaluated the effects of graded levels of dietary GRJE (0%, 0.25%, 0.5%, 0.75% and 1%) on juvenile black rockfish growth performance, non-specific immune response, and survival in a bacterial challenge test with Gram-positive *S. iniae*.

Dietary GRJE supplementation significantly improved rockfish growth (final weight, weight gain, and SGR) and feed utilization (FE and PER), even at the lowest dose (0.25% GRJE). Numerous studies have shown that dietary supplementation with various types and concentrations of ginger significantly affects growth and feed utilization in fish [1,20,21,34,35,36]. Similar to our study, Naliato et al. [19] showed that growth performance parameters of *Oreochromis niloticus* including final body weight, WG, SGR, feed intake and feed conversion ratio, and PER were responsive to the increase on dietary ginger powder supplementation, showing a dose-dependent response and a linear correlation. These authors also described that zingiberene is the most concentrated compound in the dehydrated ginger powder, terepene is known for its smell and flavor [37], and this substance improved the feed intake and feed conversion ratio, resulting in enhanced growth and PER. Ginger oils are known to enhance palatability, which may reflect improved FE and fish growth, as reported for *O. niloticus* [20]. Ginger extracts also stimulate digestive enzyme secretion in *Oncorhynchus mykiss* and *Mesopotamichthys sharpeyi*, improving growth performance through better feed utilization [38,39]. Ahmadifar et al. [40] reported an increase in enzyme activity (amylase) in fish fed 0.1–0.3% dietary ginger powder, but no effect on growth (SGR). In contrast, Cardoso et al. [20] observed improvements in *O. niloticus* growth with ginger supplementation but no differences in digestive enzyme activity (protease, lipase, and amylase).

Rockfish whole-body chemical composition was not significantly affected by the dietary GRJE concentration. This finding is consistent with our previous study, in which the proximate composition of juvenile *S. schlegelii* was not significantly affected by diet supplementation with 1% GRJE [34]. In contrast, Kim et al. [33] reported significantly higher crude protein, crude lipid, and moisture content in juvenile *S. schlegelii* fed 1% dried ginger for 8 weeks. Additionally, juvenile *Cyprinus carpio* fed varying doses of ginger extract showed significant differences in carcass composition [6]. Ginger composition is affected by the type (dry powdered plant, extract, residue), variety, agronomics, drying, and storage conditions, which may contribute to the varied effects of dietary ginger on fish [40].

Blood biochemical measurements are important aquaculture parameters that reflect fish performance and health [41,42]. In this study, plasma parameters of *S. schlegelii* were not significantly affected by dietary GRJE supplementation. Chung et al. [21] reported that increasing concentrations of ginger essential oil in *O. niloticus* diet had a negative linear effect on plasma cholesterol level but did not significantly relate to plasma glucose, triglyceride, TPs, albumin, ALT, and AST. However, *Cyprinus carpio* fed ginger extract showed increases in serum TP, albumin, and globulin content [6]. The advantageous effects of ginger supplementation on biochemical parameters indicative of promoted health have also been reported in *Lates calcarifer* [16], *Danio rerio* [40], *Huso huso* [43], and *O. mykiss* [35].

Lysozyme is compositionally expressed, synthesized, and secreted by neutrophils, monocytes, and macrophages and is considered one of the most important bacterial enzymes involved in fish immunity [44]. Lysozyme is an indispensable defense against infectious agents [45]. SOD, CAT, and GSH, which are responsible for detoxifying harmful reactive oxygen species generated during normal metabolism, are also important aspects of innate immunity in fish [45]. The SOD and CAT activity and GSH content trends observed in this study corroborate the lysozyme activity results; there was a linear increase in antioxidant enzyme activity with increasing GJRE levels. Stoilova et al. [46] demonstrated that ginger acts as an antioxidant by scavenging DPPH (1,1-diphenyl-2-picrylhydrazyl) and inhibiting the formation of secondary products from fat auto-oxidation. Additionally, ginger polyphenols have a high chelatoforming capacity with Fe^3+^, which prevents hydroxyl radical formation and lipids [46]. Masuda et al. [47] emphasized that the antioxidant activity of ginger might be due not only to radical scavenging activity, but also to its affinity with the substrate. Ahn et al. [24] reported that the ginger by-product of medicinal extraction contains polyphenol and flavonoids, which also have DPPH and ABTS (3-ethylbenzothiazoline-6-sulphonic acid) radical scavenging abilities. These findings demonstrate the benefits of GRJE dietary supplementation at levels of 0.75% and higher for sustaining and improving the oxidative status of juvenile rockfish.

Ginger-induced enhancement of the non-specific immune response was previously demonstrated for *O. niloticus* fed 1% and 1.5% ginger powder [48,49]. Similarly, Şahan et al. [50] observed that 0.5% and 1% ginger supplementation enhanced SOD and CAT activities in the liver, gills, and gut of *O. niloticus*. Significantly higher activities of SOD and CAT were observed in ginger-fed groups, notably in the 2% ginger extract supplementation group [6]. Moreover, ginger is a potent antioxidant [9], and the beneficial effects of ginger on fish antioxidant systems have been reported [1,40,51]. These effects are attributed to bioactive compounds in ginger, especially polyphenols, flavonoids, tannins, and saponins [16,52,53].

Immunostimulants increase immunocompetency and disease resistance by augmenting non-specific and specific defense mechanisms in fish [54,55,56]. In this study, dietary GRJE supplementation at 0.75–1% effectively improved disease resistance to *S. iniae*, which was also reflected in linearly positive changes in non-specific immune parameters. Similarly, dietary ginger powder administration to different fish species increased bactericidal activity against *A. hydrophila* [35], *V. harveyi* [1,16], and *A. salmonicida* [1]. Moreover, ginger powder administration significantly improved *O. niloticus* resistance against *S. agalactiae* infection [17]. Dietary ginger powder also positively impacted the treatment of Gram-positive and/or Gram-negative bacterial infections in *S. schlegelii* [33,34,57]. Lee et al. [34] reported that 1% GRJE-supplemented *S. schlegelii* groups attained a higher probability of survival against Gram-negative bacteria (*V. harveyi*) compared with groups that did not receive GRJE supplementation. These findings support the use of GRJE feed additives to reduce mortality caused by Gram-negative and Gram-positive bacterial infections.

Thus, we can conclude that, along with other ginger feed supplements, GRJE acts as a growth promoter and immunostimulant [1,6,16,21], enhancing antioxidant capacity, innate immune functions, and the overall health status of fish.

## 5. Conclusions

Dietary 0.75% GRJE supplementation is recommended as an effective and safe immunostimulatory agent for protection against bacterial diseases and the improvement of juvenile black rockfish health.

## Figures and Tables

**Table 1:** Experimental diet formulation (%, dry matter basis).

	Experimental Diets				
	GRJE0	GRJE0.25	GRJE0.5	GRJE0.75	GRJE1
Ingredients (%)					
Pollock meal	50	50	50	50	50
Fermented soybean meal	11.5	11.5	11.5	11.5	11.5
Wheat flour	27	26.75	26.5	26.25	26
Ginger residue of juice extraction (GRJE ^a^)	0	0.25	0.5	0.75	1
Fish oil	4.5	4.5	4.5	4.5	4.5
Soybean oil	4.5	4.5	4.5	4.5	4.5
Vitamin premix ^b^	1	1	1	1	1
Mineral premix ^c^	1	1	1	1	1
Choline	0.5	0.5	0.5	0.5	0.5
Nutrients (%)					
Dry matter	94.9	95.2	95.2	95.6	94.9
Crude protein	50.6	51.0	50.9	50.3	50.9
Crude lipid	14.0	14.1	13.9	13.9	13.9
Ash	8.9	8.8	9.0	8.8	9.1

^a^ Ginger residue from juice extraction supplied from Youngjin Health Food Store (Daegu, Korea); ^b^ Vitamin premix containing the following ingredients (g/kg mix) diluted in cellulose: L-ascorbic acid, 121.2; DL-α-tocopheryl acetate, 18.8; thiamin hydrochloride, 2.7; riboflavin, 9.1; pyridoxine hydrochloride, 1.8; niacin, 36.4; Ca-D-pantothenate, 12.7; myo-inositol, 181.8; D-biotin, 0.27; folic acid, 0.68; p-aminobenzoic acid, 18.2; menadione, 1.8; retinyl acetate, 0.73; cholecalciferol, 0.003; cyanocobalamin, 0.003; ^c^ Mineral premix containing the following ingredients (g/kg mix): MgSO_4_·7H_2_O, 80.0; NaH_2_PO_4_·2H_2_O, 370.0; KCl, 130.0; ferric citrate, 40.0; ZnSO_4_·7H_2_O, 20.0; Ca-lactate, 356.5; CuCl, 0.2; AlCl_3_·6H_2_O, 0.15; KI, 0.15; Na_2_Se_2_O_3_, 0.01; MnSO_4_·H_2_O, 2.0; CoCl_2_·6H_2_O, 1.0.

**Table 2:** Growth performance and feed utilization of juvenile black rockfish fed experimental diets with graded levels of GRJE for 8 weeks.

Items	Experimental Diets					SEM	Orthogonal Contrast			Regression		
	GRJE0	GRJE0.25	GRJE0.5	GRJE0.75	GRJE1		Linear	Quadratic	Cubic	Model	*p*-Value	Adj. R^2^
IBW (g)	2.2 ^a^	2.2 ^a^	2.2 ^a^	2.2 ^a^	2.2 ^a^	0.10	0.188	0.260	0.496	NR	-	-
FBW (g)	9.7 ^a^	10.5 ^b^	10.4 ^b^	10.3 ^b^	10.3 ^b^	0.35	0.030	0.009	0.034	Qd	0.016	0.497
SR (%)	96.7 ^a^	98.9 ^a^	95.6 ^a^	95.6 ^a^	98.9 ^a^	1.37	0.633	0.240	0.104	NR	-	-
WG (g/fish)	7.4 ^a^	8.3 ^b^	8.2 ^b^	8.1 ^b^	8.1 ^b^	0.36	0.050	0.010	0.034	Qd	0.021	0.474
SGR (%/day)	2.98 ^a^	3.19 ^b^	3.15 ^b^	3.13 ^b^	3.12 ^b^	0.16	0.036	0.004	0.022	Qd	0.014	0.511
FC (g/fish)	9.0 ^a^	9.0 ^a^	8.8 ^a^	8.9 ^a^	8.7 ^a^	0.34	0.127	0.682	0.686	NR	-	-
FE	0.85 ^a^	0.93 ^b^	0.93 ^b^	0.93 ^b^	0.93 ^b^	0.13	0.025	0.023	0.167	Qd	0.012	0.523
PER	1.64 ^a^	1.81 ^b^	1.82 ^b^	1.81 ^b^	1.81 ^b^	0.16	0.007	0.004	0.122	Qd	0.002	0.637

Values (means of triplicate ± SE) in the same row sharing the different superscript letter are significantly different *p* < 0.05). Abbreviations: GRJE, ginger residue from juice extraction; SEM, pooled standard error of treatment means; Adj. R^2^: adjusted R square; IBW, initial body weight; FBW, final body weight; SR, survival; WG, weight gain; SGR, specific growth rate; FC, feed consumption; FE, feed efficiency; PER, protein efficiency ratio; L, linear; Qd, quadratic; Cu, cubic; NR, no relationship.

**Table 3:** Whole-body proximate composition (%, wet weight basis) of juvenile black rockfish fed experimental diets with graded levels of GRJE for 8 weeks.

Items	Experimental Diets					SEM	Orthogonal Contrast			Regression		
	GRJE0	GRJE0.25	GRJE0.5	GRJE0.75	GRJE1		Linear	Quadratic	Cubic	Model	*p*-Value	Adj. R^2^
Moisture(%)	72.2 ^a^	72.3 ^a^	72.0 ^a^	72.4 ^a^	72.3 ^a^	0.37	0.092	0.280	0.458	NR	-	-
Crude protein (%)	16.6 ^a^	16.7 ^a^	16.9 ^a^	16.8 ^a^	16.8 ^a^	0.40	0.284	0.710	1.000	NR	-	-
Crude lipid (%)	5.9 ^a^	5.7 ^a^	5.9 ^a^	5.8 ^a^	5.7 ^a^	0.31	0.072	0.367	0.664	NR	-	-
Ash (%)	4.3 ^a^	4.1 ^a^	4.2 ^a^	4.3 ^a^	4.1 ^a^	0.30	0.554	0.506	0.766	NR	-	-

Values (means of triplicate ± SE) in the same row sharing the different superscript letter are significantly different (*p* < 0.05). Abbreviations: GRJE, ginger residue from juice extraction; SEM, pooled standard error of treatment means; Adj. R^2^: adjusted R square; L, linear; Qd, quadratic; Cu, cubic; NR, no relationship.

**Table 4:** Hematological parameters of juvenile black rockfish fed experimental diets with graded levels of GRJE for 8 weeks.

Items	Experimental Diets					SEM	Orthogonal Contrast			Regression		
	GRJE0	GRJE0.25	GRJE0.5	GRJE0.75	GRJE1		Linear	Quadratic	Cubic	Model	*p*-Value	Adj. R^2^
AST (U/L)	126.3 ^a^	155.7 ^a^	119.3 ^a^	117.0 ^a^	116.7 ^a^	2.38	0.355	0.519	0.521	NR	-	-
SLT (U/L)	32.0 ^a^	30.3 ^a^	30.3 ^a^	31.3 ^a^	31.3 ^a^	1.82	0.976	0.740	0.809	NR	-	-
T-CHO (mg/dL)	216.0 ^a^	206.7 ^a^	204.3 ^a^	208.7 ^a^	208.0 ^a^	2.65	0.572	0.417	0.627	NR	-	-
TP (g/dL)	5.0 ^a^	5.0 ^a^	5.0 ^a^	5.1 ^a^	5.2 ^a^	0.43	0.595	0.653	0.884	NR	-	-
GLU (mg/dL)	60.0 ^a^	60.0 ^a^	63.7 ^a^	59.0 ^a^	62.0 ^a^	2.20	0.852	0.902	0.803	NR	-	-

Values are means from triplicated groups of fish where the values in the same column sharing the different superscript letter are significantly different (*p* < 0.05). Abbreviations: GRJE, ginger residue from juice extraction; SEM, pooled standard error of treatment means; Adj. R^2^: adjusted R square; AST, aspartate aminotransferase; ALT, alanine aminotransferase; T-CHO, total cholesterol; TP, total protein; GLU, glucose; L, linear; Qd, quadratic; Cu, cubic; NR, no relationship.

**Table 5:** Lysozyme activity and antioxidant parameters of juvenile black rockfish fed experimental diets with graded levels of GRJE for 8 weeks.

Items	Experimental Diets					SEM	Orthogonal Contrast			Regression		
	GRJE0	GRJE0.25	GRJE0.5	GRJE0.75	GRJE1		Linear	Quadratic	Cubic	Model	*p*-Value	Adj. R^2^
Lysozyme (U/mL)	57.0 ^a^	59.2 ^a^	58.7 ^a^	62.4 ^b^	64.2 ^b^	0.95	0.000	0.369	0.785	L	0.000	0.714
SOD (U/mL)	5.3 ^a^	5.7 ^a^	5.9 ^a^	7.1 ^b^	7.7 ^b^	0.59	0.000	0.298	0.818	L	0.000	0.707
CAT (nmol/min/mL)	2.1 ^a^	2.3 ^a^	2.3 ^a^	2.6 ^b^	2.9 ^b^	0.31	0.000	0.160	0.366	L	0.000	0.740
GSH (µM)	15.2 ^a^	15.3 ^a^	15.4 ^a^	18.3 ^a^	18.8 ^b^	0.86	0.002	0.246	0.347	L	0.002	0.545

Values are means from triplicated groups of fish where the values in the same column sharing the different superscript letter are significantly different (*p* < 0.05). Abbreviations: GRJE, ginger residue from juice extraction; SEM, pooled standard error of treatment means; Adj. R^2^: adjusted R square; SOD, superoxide dismutase; CAT, catalase, GSH, glutathione; L, linear; Qd, quadratic; Cu, cubic; NR, no relationship.

## Data Availability

Data are available upon reasonable request.
